# Increased renal function decline in fast metabolizers using extended-release tacrolimus after kidney transplantation

**DOI:** 10.1038/s41598-021-95201-5

**Published:** 2021-08-02

**Authors:** Gerold Thölking, Brigitte Filensky, Ulrich Jehn, Katharina Schütte-Nütgen, Raphael Koch, Christine Kurschat, Hermann Pavenstädt, Barbara Suwelack, Stefan Reuter, Dirk Kuypers

**Affiliations:** 1 Department of Internal Medicine and Nephrology, University Hospital of Münster Marienhospital Steinfurt, 48565 Steinfurt, Germany; 2 Division of General Internal Medicine, Nephrology and Rheumatology, Department of Medicine D, University Hospital of Münster, Münster, Germany; 3 Institute of Biostatistics and Clinical Research, University of Münster, Münster, Germany; 4 Department II of Internal Medicine and Center for Molecular Medicine Cologne, Faculty of Medicine, University Hospital Cologne, Cologne, Germany; 5 Department of Nephrology and Renal Transplantation, University Hospitals Leuven, Leuven, Belgium

**Keywords:** Medical research, Nephrology

## Abstract

Fast metabolism of immediate-release tacrolimus (IR-Tac) is associated with decreased kidney function after renal transplantation (RTx) compared to slow metabolizers. We hypothesized, by analogy, that fast metabolism of extended-release tacrolimus (ER-Tac) is associated with worse renal function. We analyzed data from patients who underwent RTx at three different transplant centers between 2007 and 2016 and received an initial immunosuppressive regimen with ER-Tac, mycophenolate, and a corticosteroid. Three months after RTx, a Tac concentration to dose ratio (C/D ratio) < 1.0 ng/ml · 1/mL defined fast ER-Tac metabolism and ≥ 1.0 ng/ml · 1/mL slow metabolism. Renal function (estimated glomerular filtration rate, eGFR), first acute rejection (AR), conversion from ER-Tac, graft and patient survival were observed up to 60-months. 610 RTx patients were divided into 192 fast and 418 slow ER-Tac metabolizers. Fast metabolizers showed a decreased eGFR at all time points compared to slow metabolizers. The fast metabolizer group included more patients who were switched from ER-Tac (p < 0.001). First AR occurred more frequently (p = 0.008) in fast metabolizers, while graft and patient survival rates did not differ between groups (p = 0.529 and p = 0.366, respectively). Calculation of the ER-Tac C/D ratio early after RTx may facilitate individualization of immunosuppression and help identify patients at risk for an unfavorable outcome.

## Introduction

The calcineurin inhibitor (CNI) tacrolimus (Tac) is highly effective in preventing acute transplant rejection and is consequently recommended as first-line immunosuppressive therapy after renal transplantation (RTx)^1^. Currently, approximately 95% of RTx recipients are discharged after RTx receiving a Tac-based immunosuppressive regimen^2^.

While immediate-release Tac (IR-Tac), which is administrated twice daily, has become established over the past 2 decades, once-daily Tac formulations such as extended-release Tac (ER-Tac) and LCP-Tac are gaining traction due to convenience and higher adherence rates^3, 4^. In the current study, we focus on ER-Tac which has different pharmacokinetics (PK) compared to other Tac products. Replacement of croscarmellose in IR-Tac with ethylcellulose in ER-Tac slows down the diffusion rate of Tac, resulting in prolonged release with 90% absorption after 6–12 h^5, 6^. While one day after RTx, the mean area under the curve (AUC)_0–24_ is approximately 30% lower for ER-Tac compared with IR-Tac at comparable dosing, both formulations showed a good correlation between C_min_ and AUC_0-24_ after day 14^7^. ER- and IR-Tac have a narrow therapeutic window and high intra- and interindividual variability, therefore transplant recipients are at risk of underexposure leading to rejection or overexposure causing e.g. CNI-related toxicity^8, 9^. With this in mind, sequential therapeutic drug monitoring is standard of care, although C_min_-based dose titration is unfortunately limited in predicting individual efficacy^9, 10^.

On the one hand, non-adherence to Tac remains an underestimated problem; on the other hand, Tac metabolism and PK profiles are increasingly coming into focus^11, 12^. Once-daily drugs such as ER-Tac can improve treatment adherence, but two recent meta-analyses showed comparable clinical outcomes of ER-Tac and IR-Tac^4, 13–15^.

Recently, we introduced the C/D ratio for IR-Tac, calculated by dividing the Tac trough concentration (C) by the daily Tac dose (D), as a simple tool to estimate IR-Tac metabolism ^16^. A low C/D ratio (C/D ratio < 1.05 ng/mL · 1/mg in RTx recipients; < 1.09 ng/mL · 1/mg in liver transplant recipients (LTR)) indicates fast Tac metabolism and is associated with a decreased renal function, a higher rate of biopsy-proven CNI nephrotoxicity (CNIT), and more frequent switching to an alternative immunosuppressive regimen than a higher C/D ratio^16–21^. Moreover, a low C/D ratio is associated with higher C_2_ Tac levels and higher rejection rates despite comparable trough levels^21–23^.

Although the PK profile of ER-Tac is different from the IR-Tac profile, there are also comparable blood concentrations at certain time points (such as C_max_ or target trough levels). Therefore, the aim of the study was to evaluate whether the C/D ratio is also suitable for categorization and risk assessment or risk stratification in ER-Tac treated RTx patients^9, 24^.

## Patients and methods

This retrospective observational multicenter study included RTx recipients who underwent transplantation in Cologne and Münster, Germany and Leuven, Belgium between 2007 and 2018. Only patients with an initial immunosuppression consisting of ER-Tac (Astellas), mycophenolate, a corticosteroid, an induction therapy with basiliximab at day 0 and 4, and available C/D ratio at 3 months after transplantation were included. Pregnant women and RTx recipients younger than 18 years of age were excluded. ER-Tac was started at 0.2 mg/kg body weight q.d. with a target trough level of 8–12 ng/mL during the first 3 months, 4–10 ng/mL from month 4 to 6 and 4–8 ng/mL thereafter. Mycophenolate and corticosteroids were given according to local transplant center protocols. The transplant centers Cologne and Münster administered prednisolone and Leuven methylprednisolone.

General demographic data and information on transplantation were obtained from electronic health records of the hospitals (recipients) or from Eurotransplant (donor data). All data were anonymized before analysis. The study was approved by the local ethics committees (Ethik Kommission der Ärztekammer Westfalen-Lippe und der Medizinischen Fakultät der Westfälischen Wilhelms-Universität, No. 2014-381-f-N; Ethik Kommission des Uniklinikums Köln, protocol 14-30; Medical Ethics Committee University Hospitals Leuven, Herestraat 49-B-3000, Leuven, protocol S53364). Methods in this study were carried out in accordance with the current transplantation guidelines, the Declarations of Istanbul and Helsinki, and the International Conference on Harmonization Good Clinical Practice guidelines. All recipients gave written informed consent at the time of transplantation for recording of their clinical data and use in anonymized analyses. No organs or tissue were procured from prisoners.

**Figure 1:**
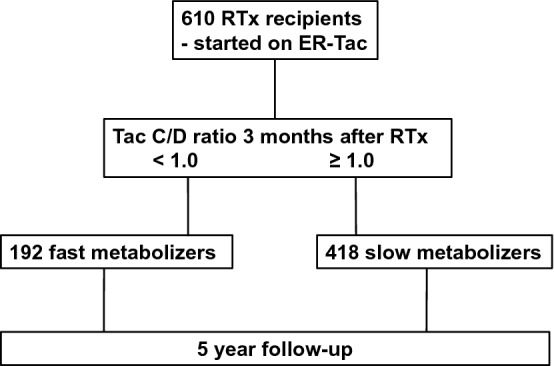
Study recruitment. 610 patients were included on the basis of taking extended-release tacrolimus (ER-Tac) in the first week after transplantation. Three months after RTx, patients were divided in fast and slow metabolizers with regards to their ER-Tac concentration to dose ratio (C/D ratio). RTx recipients were observed up to 5-years after RTx.

Patients were divided into two ER-Tac metabolism groups according to their C/D ratio 3 months after RTx (Fig. 1). The C/D ratio was calculated in analogy to previous publications with IR-Tac^16, 25^. Kidney donor profile index (KDPI) was calculated as previously published using the Organ Procurement and Transplantation Network (OPTN) online calculator^26^.

$$C/D ratio (\mathrm{ng}/\mathrm{mL }\cdot1/\mathrm{mg}) = \frac{blood ER-Tac trough level (ng/mL)}{daily ER-Tac dose (mg)}$$

A C/D ratio < 1.0 ng/mL · 1/mg at 3 months after transplantation indicated fast ER-Tac metabolizers and a C/D ratio ≥ 1.0 ng/mL 1/mg · slow metabolizers.

### Endpoints

The main aim was to investigate the evolution of renal function in the first 5 years after RTx. Therefore, the estimated glomerular filtration rate (eGFR) was calculated from serum creatinine values at day 10 (D10) and months 1, 2, 3, 6, 12, 24, 36, 48, 60 (M1-M60) after RTx. Creatinine was analyzed in a whole blood sample (enzymatic assay; Creatinine-Pap, Roche Diagnostics, Mannheim, Germany). Calculation of the eGFR was performed using the Chronic Kidney Disease Epidemiology Collaboration equation (CKD-EPI). In a first approach, eGFR values were compared between patients with a C/D ratio < 1 ng/mL · 1/mg vs. ≥ 1.0 ng/mL · 1/mg. In the next step, the influence of the M3 C/D ratio on eGFR was evaluated. Therefore, the eGFR changes from each time point to M3 were compared within each connected metabolizer group (eGFR slope) and between both groups.

The time to the occurrence of the first event of “switch of immunosuppression”, “graft failure”, and “death”, whichever occurred first, was determined as further subject of the study. “Switch of immunosuppression” was defined as the conversion from ER-Tac to any other immunosuppression. In a first step, the occurrence of “first AR” in the period RTx until M3 was analyzed of patients who received a Tac C/D ratio at M3 and were subsequently characterized as fast or slow metabolizers. In a second step, the impact of the M3 C/D ratio on further “first AR” events was assessed in a 5-year follow-up, when the time to “first AR” was investigated. Switching of immunosuppression, graft failure or death ended follow-up, so patients who switched, restarted dialysis or died without prior AR were censored at the respective date. Any “first AR” was recorded in case of AR treatment or biopsy-proven acute rejection (BPAR) and subsequent treatment. Histologic results on rejections in the transplant centers Cologne and Münster were obtained from indication biopsies only. The transplant center Leuven performed protocol biopsies 3, 12 and 24 months after RTx. The BANFF 2019 criteria were used to define BPAR. CNIT was assess by the local pathologists according to the histological patterns described in detail by Naesens et al.^27^. “Graft failure” was defined as irreversible deterioration of kidney function requiring permanent renal replacement therapy.

### Statistical analysis

Statistical analyses were performed using IBM SPSS^®^ Statistics 27 for Windows (IBM Corporation, Somers, NY, USA) and SAS software, Version 9.4 TS1M5 of the SAS System for Windows (Copyright © 2021 SAS Institute Inc., Cary, NC, USA). All p-values and confidence limits were two-sided and were intended to be exploratory, not confirmatory. Therefore, no adjustment for multiplicity was made. Exploratory p-values ≤ 0.05 were considered to be statistically noticeable.

In descriptive analysis, normally-distributed continuous variables are reported as mean ± standard deviation and not normally-distributed continuous variables as median (25% quantile–75% quantile, IQR). Absolute and relative frequencies are given for categorical variables. Metabolism groups were compared using Welch’s t-tests for normally-distributed data, Mann–Whitney U-tests for skewed-distributed data, and Fisher’s exact tests for categorical variables. Comparison of the eGFR changes within each metabolism group was performed using Wilcoxon’s signed-rank tests. Boxplots were used for graphical representation.

In order to model renal function (eGFR) over time adjusted for co-variables and dropouts over time, a multivariable linear mixed model was fitted. Recipient’s age, European Senior Program (ESP) (yes/no), the main effects of time (3, 6, 12, 24, 36, 48, 60 months) and metabolizer group (fast/slow), and the interaction between time and group were included as influencing variables. Repeated measurements of each patient were modeled using SAS PROC MIXED by fitting a marginal linear mixed model with an unstructured variance–covariance matrix for the residuals with patient as subject and the order given by time. The empirical sandwich estimator was applied. Missing values were treated as missing at random. Results are reported as least square estimates with corresponding 95% confidence interval (CI), and p-values from the Wald test.

Event rates of the “first AR”, of the component endpoint “switch of the immunosuppression”, “graft failure” and “death as first event” (whatever occurred first), as well as of overall survival were estimated using the Kaplan–Meier method with 95% CI using log-transformation. Patients without an event were censored at their last visit date. In the analysis of “first AR”, patients who switched immunosuppression, showed a graft failure or died without prior AR were additionally censored at the respective date. All time-to-event endpoints started at 3 months after RTx. Consequently, AR and graft failures which occurred until 3 months were not considered in the analysis and patients were excluded from these analyses. Hazard ratios (HR) and 95% CI are given to quantify the effect of fast versus slow metabolizers. A competing risk approach was used to estimate the effect on each component of the combined endpoint. The impact of fast vs. slow metabolizer on the components was thus estimated using Fine and Gray's model leading to subdistribution hazard ratios (sub-HR). Cumulative incidence was estimated using the Aalen-Johansen estimator. Gray's k-sample test was applied to compare the cumulative incidence of the corresponding event type. Additionally, the cause-specific hazard (csH) for each component of the competing event type was compared between the metabolizer groups via cause-specific hazard ratios (csHR) using the methods by Prentice (*results not shown*).

## Results

**Figure 2:**
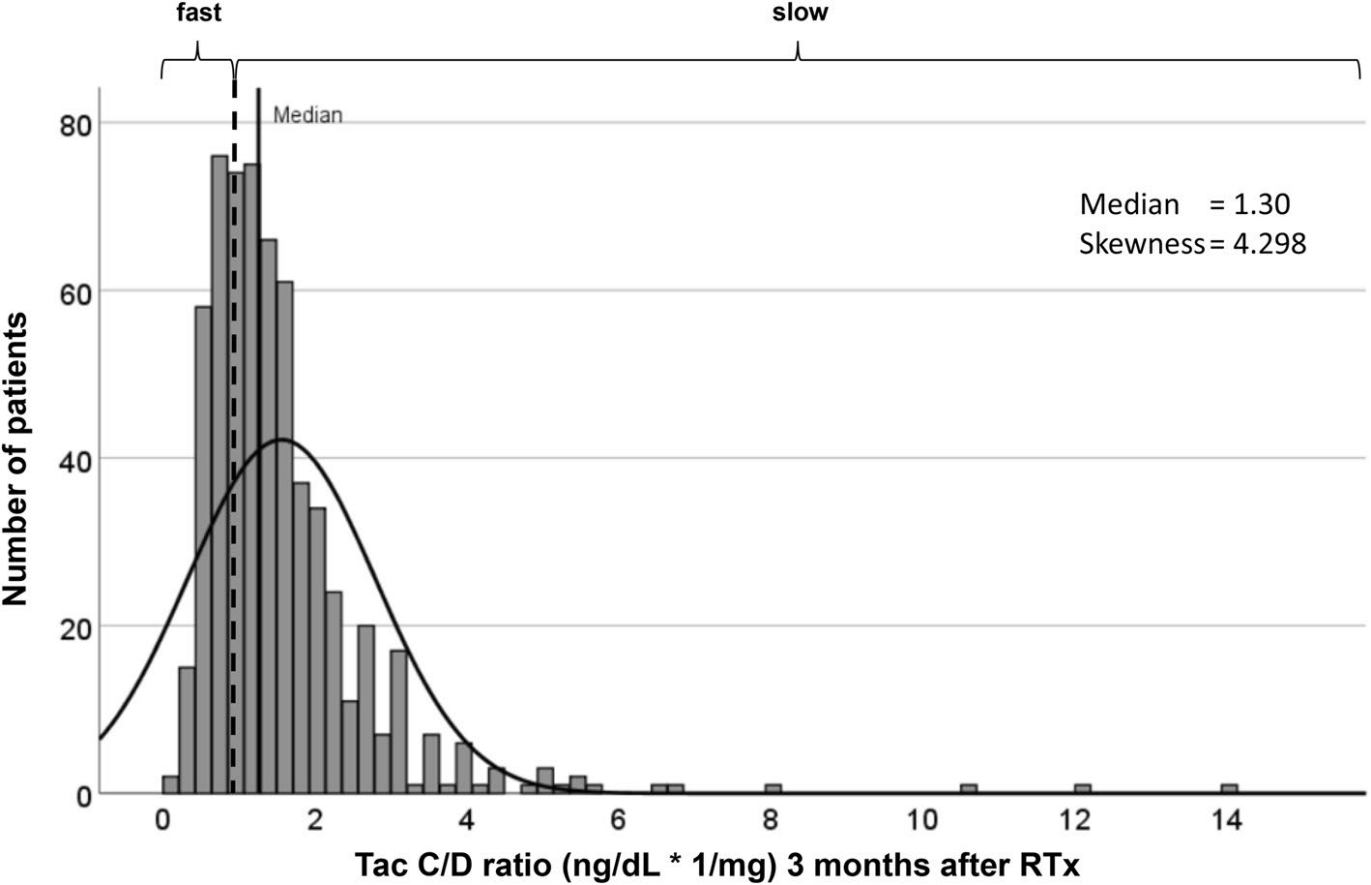
Empirical distribution of the patients in terms of their concentration to dose ratio (C/D ratio) three months after RTx. Fast ER-Tac metabolizers were defined by a C/D ratio < 1 ng/mL · 1/mg, and slow metabolizers had a C/D ratio ≥ 1 ng/mL · 1/mg.

We included and analyzed 610 RTx recipients in a 5-year follow-up. Using the categorization of the C/D ratio of < 1.0 ng/mL · 1/mg and ≥ 1.0 ng/mL · 1/mg at 3 months after RTx, the cohort was divided into approximately 1/3 (n = 192) fast and 2/3 (n = 418) slow metabolizers, similar to previous studies (Figs. 1 and 2)^16, 21^.

The RTx cohort showed a skew distribution of the ER-Tac C/D ratio at 3 months with a median of 1.3 (IQR 0.86–1.88) ng/mL · 1/mg.

Table 1 shows the baseline characteristics of the patients. Fast ER-Tac metabolizers were younger (p = 0.003) although more patients within the ESP (p = 0.006) received a graft, albeit with low absolute ESP numbers. There were no other noticeable differences in patient characteristics.

**Table 1:** Patients characteristics.

	Fast metabolizers n = 192	Slow metabolizers n = 418	p-value
Body weight (kg)	74.7 ± 14.5	73.6 ± 14.8	0.512^a^
Height (m)	1.71 ± 0.10	1.70 ± 0.09	0.508^a^
BMI (kg/m^2^)	24.7 (22.1–27.4)	24.5 (22.6–27.1)	0.810^c^
Age (years)	52.5 ± 14.3	56.0 ± 11.8	0.003^a^
Sex (m/f, %)	120 (62.5%) / 72 (37.5%)	266 (64%) / 152 (36%)	0.787^b^
Living donor transpl.	14 (7.3%)	23 (5.5%)	0.465^b^
Cadavaric donor transpl.	178 (92.7%)	395 (94.5%)	
HBD	155 (80.7%)	340 (81.3%)	0.728^b^
NHBD I	19 (9.9%)	42 (10%)	
NHBD II	1 (0.5%)	0	
NHBD III	17 (8.9%)	35 (8.4%)	
NHBD IV	0	1 (0.2%)	
ESP transpl.	8 (4.2%)	3 (0.7%)	0.006^b^
ABO-incompatible transpl.	0	2	–
Cold ischemia time (h)	12.1 ± 5.8	12.5 ± 5.8	0.525^a^
Delayed graft function	32 (16.7%)	77 (18.4%)	0.650^b^
KDPI	49.0 ± 25.8	47.0 ± 25.4	0.402^a^
Warm ischemia time (min)	35 (30–41)	35 (30–45)	0.262^c^
**HLA MM**			
0	8 (4.2%)	28 (6.7%)	0.393^b^
1–3	117 (60.9%)	240 (57.4%)	
4–6	59 (30.7%)	140 (33.5%)	
PRA > 20%	13/177 (6.8%)	27/394 (6.5%)	0.860^b^
**Combined transpl.**			
+ Pancreas	4 (2.1%)	12 (2.9%)	0.695^b^
+ Liver	3 (1.6%)	3 (0.7%)	
+ Heart	0	1 (0.2%)	
Non-combined	185 (96.4%)	402 (96.2%)	
Donor characteristics			
Donor age (years)	49.7 ± 16.0	49.0 ± 15.1	0.601^a^
Donor sex (m/f) %	82 (52%) / 76 (48%)	196 (51%) / 188 (49%)	0.925^b^
Donor height (m)	1.71 ± 0.09	1.72 ± 0.09	0.326^a^
Donor weight (kg)	74.1 ± 14.8	74.6 ± 14.4	0.718^a^
Donor BMI (kg/m^2^)	24.7 (22.6–26.6)	24.8 (22.5–26.8)	0.936^c^

Data presented as mean ± standard deviation or median (25% quantile-75% quantile), or absolute and relative frequencies.

*BMI* body mass index, *transpl.* transplantation, *HBD* heart-beating donors, *NHBD* non-heart-beating donors (only from the Leuven-cohort), *ESP* European Senior Program, *KDPI* kidney donor profile index, *HLA MM* human leucocyte antigen mismatch, *PRA* panel reactive antibodies.

^a^Welch's t-test.

^b^Fisher's exact test.

^c^Mann–Whitney U test.

### Immunosuppression

Compared to slow metabolizers, the group with a C/D ratio < 1.0 ng/mL · 1/mg received higher ER-Tac doses at 1, 3 and 6 months after RTx (all p < 0.001), and even had slightly lower Tac trough levels at month 1 and 3 (both p < 0.001; Table 2). Median Tac blood concentrations were within the target trough level at all three time points. In the Leuven study center, fast metabolizers received slightly more methylprednisolone than slow metabolizers at month 1 after RTx (12 (IQR 9.5–16.0) mg vs. 12 (IQR 8.0–12.0) mg; p = 0.006).

**Table 2:** Doses, Tac trough level, Tac C/D ratio.

	Fast metabolizers n = 192	Slow metabolizers n = 418	p-value
**1 month after RTx**			
Tac daily dose (mg)	16 (12.0–21.0)	9 (7–12)	< 0.001^a^
Tac trough level (ng/mL)	11.0 (9–13)	12.1 (10.2–15)	< 0.001^a^
Tac C/D ratio	0.69 (0.52–0.91)	1.4 (1.0–1.89)	< 0.001^a^
Prednisone dose	15 (12.5–20)	15 (10.6–20)	0.940^a^
Methylprednisolone	12 (9.5–16)	12 (8–12)	0.006^a^
**3 months after RTx**			
Tac daily dose (mg)	14 (11–18)	7 (5–9)	< 0.001^a^
Tac trough level (ng/mL)	10.0 (8.0–11.0)	11.0 (9.3–13.0)	< 0.001^a^
Tac C/D ratio	0.72 (0.54–0.83)	1.60 (1.25–2.18)	< 0.001^a^
Prednisone dose	10 (5–15)	5 (5–7.5)	0.117^a^
Methylprednisolone	4 (4–4)	4 (4–4)	0.229^a^
**6 months after RTx**			
Tac daily dose (mg)	11 (9–15)	6 (4–7.4)	< 0.001^a^
Tac trough level (ng/mL)	9.9 (7.1–11.9)	9.4 (6.0–14.0)	< 0.916^a^
Tac C/D ratio	0.89 (0.63–1.08)	1.69 (1.29–2.33)	< 0.001^a^
Prednisone dose	5 (2.5–10)	5 (1.3–5)	0.172^a^
Methylprednisolone	4 (4–4)	4 (4–4)	0.835^a^
**Initial co-immunosuppression**			
Mycophenolate mofetil, n (%)	190 (99.3%)	415 (99%)	0.652^b^
Mycophenolate sodium, n (%)	2 (0.7%)	3 (1%)	

Data presented as mean ± standard deviation or median (25% quantile-75% quantile), or absolute and relative frequencies.

*Tac* tacrolimus, *C/D* concentration to dose.

^a^Mann–Whitney U test.

^b^Fisher’s exact test.

### Renal function

**Figure 3:**
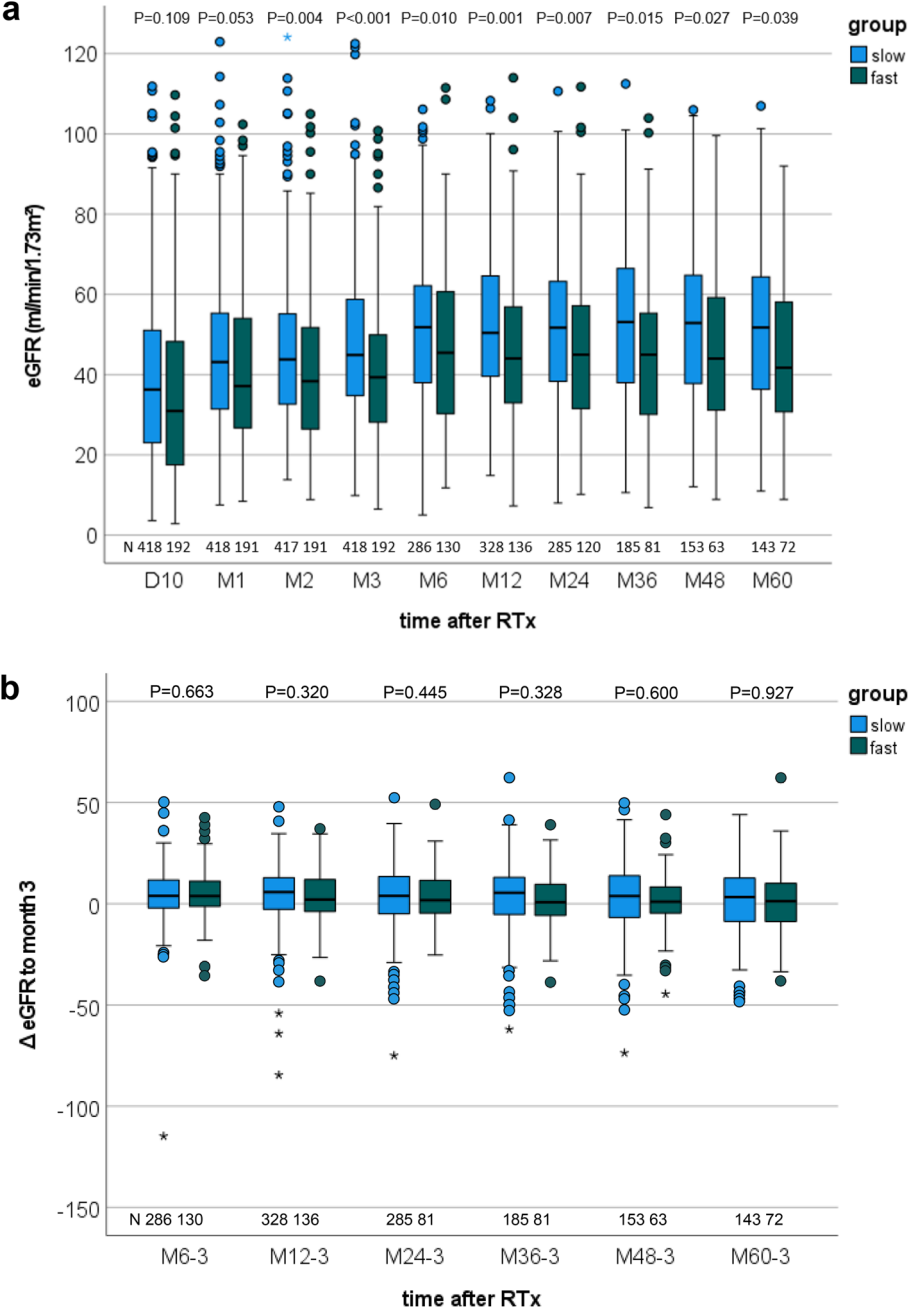
Boxplots of the renal function. Fast ER-tacrolimus metabolizers had a reduced estimated glomerular filtration rate (eGFR) as early as 10 days to 60 months (M60) after renal transplantation (RTx) compared with slow metabolizers **(a)**. Comparison of the eGFR change (ΔeGFR) from subsequent time points to M3 (Mx-3) showed no differences between metabolizer groups **(b)**. P-values are from Welch’s t-test. *D* day, *M* month.

As early as D10, renal function of the fast ER-Tac metabolizers was slightly decreased compared to the slow metabolizers and reached a noticeable difference at M2 (p = 0.004). This difference persisted over the entire time period. (Fig. 3a). In a further step, we analyzed whether the M3 C/D ratio had an influence on the subsequent eGFR change. In contrast to the different ΔeGFR development from D10 to M3 after RTx (p = 0.0362), the subsequent ΔeGFR changes from the M3 eGFR did not differ between both groups (Fig. 3b). However, 5 years after RTx the eGFR in patients with a C/D ratio < 1 ng/mL · 1/mg was lower than in patients with ≥ 1 ng/mL · 1/mg (45.6 ± 19.4 vs. 51.5 ± 19.6 mL/min/1.73 m^2^; p = 0.039).

The results in the linear mixed model were similar to the univariate analyses (Table 3). The mean eGFR in fast metabolizers was 7.5 (95% CI 4.3–10.6, p < 0.001) mL/min/1.73 m^2^ lower pooled over all time points (M3–M60). eGFR changed in the whole collective from M3 (p < 0.001) and the changes between both metabolism groups were slightly different (interaction term, p = 0.037). Age at RTx (p < 0.001) had a further potential influence on the renal function. Despite the small p-value the additional effect of age was quite low. Per year of age older the eGFR value declined by -0.4 (95% CI − 0.5 to − 0.3) mL/min/1.73 m^2^. The influence of transplantation in the ESP indicated only a trend (p = 0.084). In these patients, the eGFR was − 5.7 (95% CI − 12.2 to 0.8) mL/min/1.73 m^2^ lower than in patients who received an organ in the regular allocation system.

**Table 3:** Renal function, eGFR (linear mixed model).

Model-based estimates					
		Mean eGFR	Lower 95% confidence limit	Upper 95% confidence limit	p-value
ESP transplantation	Yes vs. no	−5.73	−12.23	0.77	0.084
Age at RTx	x vs. x − 1 years	−0.36	−0.46	−0.26	< 0.001
Effect of metabolism group combined over all time points	Fast vs. slow	−7.5	−10.6	−4.3	< 0.001
Effect of time combined over both metabolism groups					< 0.001
Interaction term of ER-Tac metabolism groups × time points					0.037
**Least square estimates of the mean difference between fast and slow metabolizer at different time points (combination of main and interaction effects of tacrolimus metabolism group and time points)**					
At 3 months	Fast vs. slow	−7.62	−10.70	−4.55	< 0.001
At 6 months	Fast vs. slow	−6.98	−10.55	−3.42	< 0.001
At 12 months	Fast vs. slow	−8.51	−11.85	−5.18	< 0.001
At 24 months	Fast vs. slow	−8.22	−11.69	−4.75	< 0.001
At 36 months	Fast vs. slow	−9.04	−12.83	−5.25	< 0.001
At 48 months	Fast vs. slow	−7.07	−11.20	−2.95	< 0.001
At 60 months	Fast vs. slow	−4.80	−9.03	−0.56	0.027
**Least square estimates of the mean change between the time points for each metabolism group (combination of main and interaction effects of tacrolimus metabolism group and time points)**					
Fast metabolizer	6 vs. 3 months	5.18	3.22	7.13	< 0.001
	12 vs. 3 months	4.08	2.11	6.05	< 0.001
	24 vs. 3 months	2.92	0.76	5.09	0.008
	36 vs. 3 months	2.16	−0.27	4.60	0.082
	48 vs. 3 months	2.45	−0.49	5.40	0.103
	60 vs. 3 months	3.87	0.84	6.89	0.012
Slow metabolizer	6 vs. 3 months	4.54	3.09	5.98	< 0.001
	12 vs. 3 months	4.97	3.57	6.37	< 0.001
	24 vs. 3 months	3.52	1.86	5.18	< 0.001
	36 vs. 3 months	3.58	1.55	5.60	< 0.001
	48 vs. 3 months	1.90	−0.24	4.04	0.082
	60 vs. 3 months	1.04	−1.26	3..33	0.375

Results of the linear mixed model. Selected parameter estimates and least square means for estimated glomerular filtration rate (eGFR) (mL/min/1.73 m^2^) are shown. P-values are from Wald tests. Repeated measurements for each patient were modelled using SAS PROC MIXED by fitting a marginal linear mixed model with an unstructured variance–covariance matrix for the residuals with patient as subject and the order given by time.

*ESP* European Senior Program, *RTx* renal transplantation.

### Time-to-event endpoints

**Figure 4:**
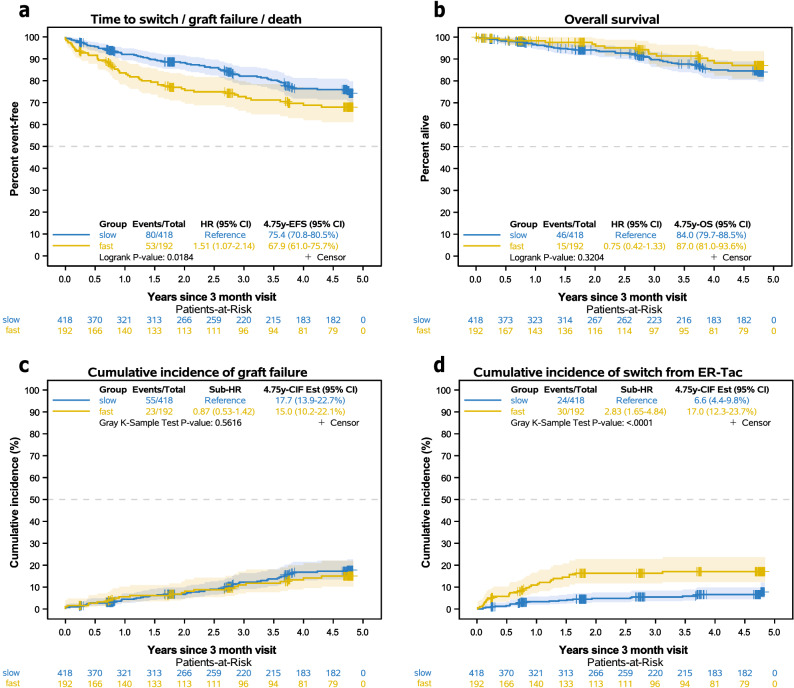
Kaplan–Meier curves of the composite endpoint “switch from ER-Tac”, “graft failure”, or “death” as first event by metabolism group starting from three months after RTx **(a)**. Overall survival is shown in **(b)**. Cumulative incidence of the “graft failure” component **(c)** and “switch from ER-Tac” **(c)** of the composite endpoint. Since only one death without prior switch or graft failure occurred in the slow metabolism group, the curves were not drawn. The impact of fast vs. slow metabolizer was thus estimated using Fine and Gray's model leading to subdistribution hazard ratios (sub-HR) on the components of the composite endpoint. Cumulative incidence was estimated using the Aalen-Johansen estimator. Gray's k-sample test was applied to compare the cumulative incidence of the corresponding event type. The combined endpoint “switch/graft failure/death” showed more events in the fast metabolizer group. Competing risk analysis revealed that “switch from ER-Tac” occurred more frequently in fast than in slow metabolizers**,** but no differences were found in regards to graft failure or overall survival.

**Figure 5:**
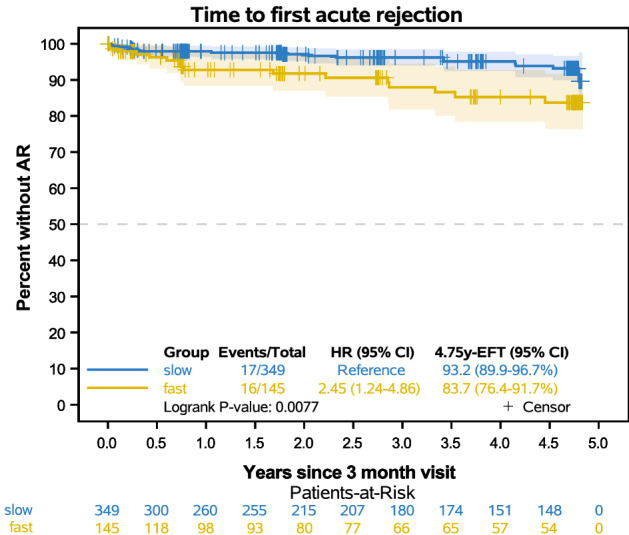
Time to “first acute rejection” (AR) from 3 months after transplantation. Patients who switched immunosuppression, showed a graft failure or died without prior AR were censored at the respective date. Patients with an AR between transplantation and 3 months were excluded (n = 116). Fast ER-Tac metabolizers showed more first rejections compared to slow metabolizers within 5-years after transplantation.

Consideration of the combined endpoint of switch/graft failure/death showed that fast metabolizers had more and earlier events than slow metabolizers over the 5-year follow-up (hazard ratio 1.51 (95% CI 1.07–2.14), Fig. 4a). Competing risk analysis indicated that crucial to these differences was a higher cumulative incidence of fast metabolizers who were switched from ER-Tac to other immunosuppression (p < 0.0001, Table 4, Fig. 4d). In detail, more fast metabolizers were switched from ER-Tac to IR-Tac (p = 0.002) or everolimus (p = 0.021). The main reasons for the change were CNIT (< 0.001) and large variations in Tac trough concentrations (p = 0.036) compared to slow metabolizers. The cumulative incidence of the component “graft failure” of the combined endpoint switch/graft failure/death was not different between the groups (p = 0.562, Fig. 4c). One slow metabolizer died without prior switch or graft failure. A difference in overall survival was not observed (p = 0.320, Fig. 4b). However, analysis of “first AR” events (after M3) showed that fast ER-Tac metabolizers developed more events compared to slow metabolizers (p = 0.0077, Fig. 5, Table 5). In a further analysis, no differences were found in the histological AR subtypes. Considering the period from RTx to M3, fast metabolizers also suffered more frequently from “first AR” than slow metabolizers (24.5% vs. 16.5%; p = 0.026, Table 5).

**Table 4:** Switch from ER-Tac to another immunosuppression.

	Fast metabolizers n = 192	Slow metabolizers n = 418	p-value
Switch from ER-Tac between 3 months and 5 years from RTx (events, 5 year-cumulative incidence, 95% CI)	30 (17.0 [12.3–23.7]%)	24 (6.6 [4.4–9.8]%)	< 0.0001^a^
**Switched to**			
IR-Tac	8	2	0.002^b^
LCP-T	1	0	0.315^b^
Everolimus	11	8	0.021^b^
ciclosporin A	10	14	0.270^b^
**Reason for switch**			
CNIT	23	16	< 0.001^b^
Large Tac level variation	4	1	0.036^b^
NODAT	1	3	–
BKVN	1	1	–
Malignancy	0	1	–
NODAT + CNIT	0	1	–
BKVN + CNIT	1	0	–
BKVN + CMV	0	1	–

Cumulative incidence was estimated using the Aalen-Johansen estimator.

*IR-Tac* immediate-release tacrolimus, *LCP-T* LCP-tacrolimus, *CNIT* calcineurin inhibitor toxicity, *NODAT* new onset diabetes after transplantation.

^a^Gray k-sample test.

^b^Fisher's exact test.

**Table 5:** First acute rejection, graft failure and death.

	Fast metabolizers n = 192	Slow metabolizers n = 418	p-value
First acute rejection (from RTx to 3 months after RTx)	47 (24.5%)	69 (16.5%)	0.026^a^
First acute rejection between 3 months and 5 years from RTx (events, 5 year-Est, 95% CI)	16/145 16.3% (8.3–23.6%)	17/349 6.8% (3.3–10.1%)	0.008^b^
**Type of first acute rejection (from 3 months to 5 years after RTx)**			
ABMR	0	2	0.095^a^
TCMR	7	10	
Borderline	9	4	
borderline + ABMR	0	1	
Graft failure as first event between 3 months and 5 years from RTx (events, 5 year-cumulative incidence, 95% CI)	23 15% (10.2–22.1%)	56 17.7% (13.9–22.7%)	0.562^c^
**Reasons for graft failure**			
Chronic allograft rejection	5	5	0.240^a^
Glomerulonephrtis recurrence	2	1	
BKVN	1	4	
Infection	2	11	
Allograft ischemia/renal artery complication	2	4	
Perirenal hematoma	1	0	
Nephrocalcinosis	0	1	
Death with functioning allograft	10	30	
Death 3 months and 5 years from RTx (events, 5 year-Est, 95% CI)	15 13.0% (6.4–19.0%)	47 16% (11.5–20.3%)	0.320^b^
**Reasons for death**			
Cardiovascular	2	5	0.776^a^
Malignancy	2	12	
Infection	3	13	
Encephalopathia	0	1	
Mayor bleeding	1	1	
Intoxication	0	1	
Trauma	0	1	
Euthanasia^A^	0	1	
Unknown	7	12	

Cumulative incidence was estimated using the Aalen-Johansen estimator.

*Est* = 1 − Kaplan–Meier estimator, *ABMR* antibody mediated rejection, *TCMR* T-cell mediated rejection, *BKVN* BK virus nephropathy, *RTx* renal transplantation.

^A^Onepatient from the Belgian cohort.

P-values: ^a^ Fisher’s exact test; ^b^Logrank test; ^c^Gray k-sample test.

## Discussion

Because IR-Tac and ER-Tac have comparable safety and efficacy, we hypothesized that a C/D ratio-guided analysis would be able to stratify patients treated with ER-Tac analogously to patients treated with IR-Tac with respect to their Tac metabolism.

Previous analysis of IR-Tac metabolism groups after RTx and liver transplantation (LTx) defined by a C/D ratio cut-off showed repeatedly and consistently worse renal outcomes in fast metabolizers compared to slow metabolizers^16, 17, 21, 22, 28^. Other studies without C/D ratio calculation, but dose and trough data pointed into the same direction^29, 30^. Interestingly, even studies who included patients with IR-Tac and ER-Tac observed comparable outcomes to pure IR-Tac studies^18, 19^. An analysis of the Korean Organ Transplantation Registry did not provide information regarding the used Tac formulation but observed the same findings^31^. Further evidence comes from PK profile analyses and assumptions. When analyzing the PK profiles of RTx patients treated with ER-Tac, one can observe the same mixed PK pattern with different profile types (probably related to the Tac metabolic type of the patients) as in IR-treated RTx recipients^32, 33^. Interestingly, the C_max_ was also not significantly different or only slightly lower than that of patients treated with IR-Tac and was comparable in healthy subjects^34, 35^. This is important, as we found high C_max_ associated with a higher degree of CNIT^22^. Thus, there was evidence from the literature suggesting a similar C/D ratio-dependent effect in IR-Tac and ER-treated patients.

We have calculated a C/D ratio cutoff of 1.0 ng/mL · 1/mg for the differentiation of metabolism types in ER-Tac treated patients. This fits well with the previously calculated cut-offs 1.05 ng/mL · 1/mg for IR-treated RTx and 1.09 ng/mL · 1/mg for LTx patients^16, 17, 21^. In addition, the TOMATO study, which included a hybrid cohort of 1029 IR- and ER-Tac patients, and which also used a C/D cutoff of 1.05 ng/mL · 1/mg, found that, on the one hand, the C/D ratio was stable between months 6–12 after RTx. On the other hand, a hazard ratio of 2.25 could be calculated for death-censored graft loss in patients with a C/D ratio < 1.05 ng/mL · 1/mg in contrast to the *CYP3A5* genotyping which was not predictive^18^. Unfortunately, the authors had no data on eGFR in the follow-up. A reduced survival as well as increased incidence of graft failure in fast metabolizers is consistent to our IR-Tac data^21^, in contrast, we could not observe the same differences in the present study (Fig. 4b,c). This may be related to the fact that during follow-up, 17.0% of patients in the fast ER-Tac metabolizer group were switched to an alternative immunosuppressive regimen, compared to only 6.6% in the slow metabolizer group (p < 0.0001, Table 4, Fig. 4d). The main reason for switching from ER-Tac was CNIT, which was observed more frequently in the group with the C/D ratio < 1.0 ng/mL · 1/mg. This is in congruence with our previous studies on IR-Tac and the observation that C_max_ could be critical for its development^16, 21, 22, 36^. IR- and ER-Tac show similar PK in terms of C_max_ and their narrow therapeutic window. Accordingly, adverse effects such as CNIT are common and may occur in similar frequency, especially in fast metabolizers who have higher C_max_ than slow metabolizers—at least in IR-Tac treated patients^22^. One limitation of our present study is that we do not have C_max_ data. Interestingly, the major difference in eGFR between both groups developed within 3 months after RTx. We already knew from former studies that CNIT can occur early after RTx, especially in patients treated with high Tac dosages^29, 37^. Recently, in de novo ER-Tac-treated patients, it was shown that a C/D ratio < 1.05 ng/mL · 1/mg and the presence of interstitial fibrosis and tubular atrophy (IF/TA) in a biopsy 3 months after RTx were associated with future IF/TA progression^38^. Of note, the authors observed that unlike Tac trough levels, intra-patient variability, or time below the therapeutic range, just a low C/D ratio was associated with IF/TA progression. This is congruent with data from Egeland et al. who identified fast Tac metabolism as a risk factor for the development of IF/TA^39^. It is important to note in this context that both Tac formulations have high intra- and inter-individual coefficients of variation for AUC, C_min_ and C_max_^40, 41^. Recently, this high intra-patient variability (IPV) in Tac trough levels especially early after RTx has been associated with rejection, de novo donor-specific antibodies (DSA), progressive IF/TA and reduced graft survival^42^. One explanation could be that patients with high IPV are outside the therapeutic range for extended periods of time since e.g. a single missed Tac dose can greatly affect exposure especially in recipients with fast metabolism^43^. It has been argued that switching patients from IR-Tac to ER-Tac may be beneficial in terms of IPV, but the data remain inconclusive and clinically limited^44, 45^. Consistent with these observations, patients with a C/D ratio < 1.0 ng/mL · 1/mg suffered more AR events than patients with a higher C/D ratio (Table 5, Fig. 5). Similar observations have been made in patients with IR-Tac-treated patients^21, 23^.

Our study has limitations. Since this is a retrospective study, it can only generate new hypotheses. Moreover, we did not assess C_max_ and coefficient of variation/IPV in our patients. Therefore, we can only assume effects of fast ER-Tac metabolism (low C/D ratio) of renal outcomes. Further, more patients in the group with a C/D ratio < 1.0 ng/mL · 1/mg were switched mainly due to side effects to an alternative immunosuppression. Thus, we cannot rule out completely that this might have affected the outcomes. However, since we have previously shown that a conversion from Tac to everolimus can be safely done in selected patients with e.g. CNIT this could have stabilized some fast metabolizers and reduced the difference in eGFR loss^36^. Donor factors may influence graft outcome. Unfortunately, donor eGFR data are not available to us. However, since the donors did not differ significantly between the groups with respect to various factors such as age, BMI, and KDPI, and the rate of DGF was comparable, we do not expect major differences in the eGFR of the donors.

## Conclusion

We conclude from our data that patients treated with ER-Tac who have a C/D ratio < 1.0 ng/mL · 1/mg showed a decreased eGFR, especially early after RTx. This was mainly associated with CNIT and a higher rate of first AR, leading to more frequent changes in immunosuppression in these patients. Thus, this group of patients has an increased risk of developing a worse outcome after RTx and therefore deserves special attention. Because it is very simple and without cost, we propose to calculate the C/D ratio early after RTx in patients treated with ER-Tac. Switching rapid ER-Tac metabolizers to other Tac formulations, such as LCP-Tac, or other immunosuppressive agents, such as belatacept or everolimus, is safe and may be beneficial, taking into account the immunologic and metabolic profile of the individual.

## Data Availability

All authors agree with the all publication's requirements for sharing materials.
